# Cdc-Like Kinases (CLKs): Biology, Chemical Probes, and Therapeutic Potential

**DOI:** 10.3390/ijms21207549

**Published:** 2020-10-13

**Authors:** Paula Martín Moyano, Václav Němec, Kamil Paruch

**Affiliations:** 1 Department of Chemistry, Faculty of Science, Masaryk University, Kamenice 5, 625 00 Brno, Czech Republic; 242772@mail.muni.cz (P.M.M.); nemecvasek2@gmail.com (V.N.); 2 International Clinical Research Center, Center for Biomolecular and Cellular Engineering, St. Anne’s University Hospital in Brno, 602 00 Brno, Czech Republic

**Keywords:** CLK, cdc-like kinase, inhibitor, chemical biology, tool compound, chemical probe, T3, SGC-CLK-1, MU1210, SM08502

## Abstract

Protein kinases represent a very pharmacologically attractive class of targets; however, some members of the family still remain rather unexplored. The biology and therapeutic potential of cdc-like kinases (CLKs) have been explored mainly over the last decade and the first CLK inhibitor, compound SM08502, entered clinical trials only recently. This review summarizes the biological roles and therapeutic potential of CLKs and their heretofore published small-molecule inhibitors, with a focus on the compounds’ potential to be utilized as quality chemical biology probes.

## 1. Introduction

Protein kinases play pivotal roles in nearly every aspect of cellular function [1]. Chemically, they transfer the *γ*-phosphate group from nucleoside triphosphate (typically ATP or GTP) to the hydroxyl group of serine, threonine, or tyrosine residues of the physiological substrate. Once the substrate is phosphorylated, it is released from the kinase and the remaining nucleoside diphosphate is recycled. The transfer of electrostatically charged phosphate group to an amino acid side chain of the target protein is often accompanied with conformational changes of the protein; thus, phosphorylation can act as a molecular “on-off” switch that strongly increases or decreases the enzymatic activity and/or the stability of the phosphorylated protein [2,3]. It has been estimated that about up to 30% of all human proteins can be phosphorylated by kinases [4,5].

Phosphorylation of proteins can lead to activation or deactivation of signal-transduction pathways that are important in various biological processes such as metabolism, transcription, cell cycle progression, cell movement, apoptosis, and differentiation. Over the past three decades, the protein kinase family has emerged as one of the most important classes of drug targets because mutations and dysregulations of protein kinases are frequently involved in the initiation and progression of cancers and other diseases [4,6,7,8,9].

Some kinases (e.g., BCR-ABL, BTK, AKT, VEGFR, etc.) have been established as attractive targets for pharmacological inhibition for a relatively long time [10,11] while others, with the increasing knowledge of the cellular mechanisms, have come to the forefront only recently [11,12,13,14].

The kinase-driven cellular processes include also alternative and constitutive splicing, which are pivotal for gene expression [15,16]. Alternative splicing gives rise to higher complexity and diversity of transcriptome via the production of alternatively spliced mRNAs, which is consequently mirrored by the higher diversity of the proteome [16,17]. Aberrant splicing is responsible for the inaccurate translation of genetic information into the structure of proteins, which has often a negative impact on various cellular processes [18,19,20,21]. The splicing process is, to a large extent, regulated by protein kinases that phosphorylate splicing factors—central components of the spliceosome, for example, serine/arginine-rich proteins (SR proteins)—and less directly also by other kinases involved in cellular signaling pathways [19,22].

SR proteins are splicing factors that interact simultaneously with RNA (via the RNA recognition motif RRM) and other proteins (via the RS domain containing arginine/serine residue repeats). They are employed in cellular processes such us constitutive and alternative pre-mRNA splicing, mRNA nuclear export, nonsense-mediated decay, and mRNA translation [23,24,25,26]. As summarized in recent reviews [16,19,22,27], SR-protein phosphorylation can be executed by SR protein kinases (SRPKs), topoisomerase 1 (TOP1), protein kinase B (PKB/AKT), NIMA-related kinase2 (NEK2), PRP4 kinase (PRP4K), dual-specificity tyrosine phosphorylation-regulated kinase 1A (DYRK1A) [28,29], cAMP-dependent protein kinase (PKA) [30,31], and by the family of cdc-like kinases (CLKs), which are the subject of this review.

Other kinases involved in the regulation of splicing include MAPKs (acting via the RAS-RAF-MEK-ERK signaling cascade), tyrosine kinases (BRK, FYN, SRC, c-ABL), Fas-activated serine/threonine kinase (FAST) [32], and possibly also AURKA [33]. Those kinases can influence the splicing via phosphorylation of splicing factors such as SAM68 (e.g., RAS-RAF-MEK-ERK cascade) [34] or more indirectly via phosphorylation of their upstream effectors.

Despite significant scientific progress in the area over the past decade, the biology of CLKs has not been completely elucidated and, particularly, the roles of individual CLK isoforms remain largely unexplored. These questions could be, at least partially, addressed by the utilization of the recently discovered highly selective tool compounds (quality probes) that are summarized in this review. 

## 2. Classification and Structure of CLKs

**Figure 1:**
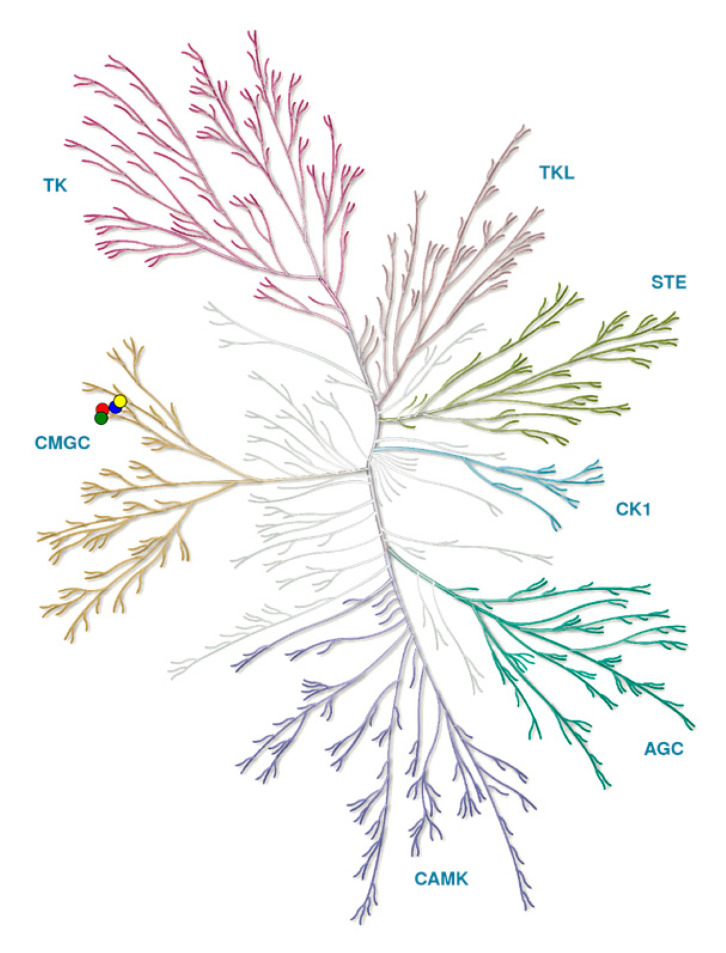
Kinome tree with CLKs marked by circles: CLK1 (blue), CLK2 (red), CLK3 (green), CLK4 (yellow). Source: *KinMap* software. Illustration reproduced courtesy of Cell Signaling Technology, Inc. (www.cellsignal.com).

Protein kinases form a large family of more than 500 proteins with common characteristics that can be clustered into phylogenetic groups (Figure 1) [35].

The group CMGC (named after initials of some key members) includes several subgroups: Cyclic Dependent Kinases (CDKs), Mitogen-Activated Protein Kinases (MAPKs), Glycogen Synthase Kinases (GSKs), and CLKs, first reported in 1991 [36,37].

Overall, CMGC is a large and well-conserved group of kinases that have pivotal roles in cell cycle regulation and intracellular signal transduction. 

**Figure 2:**
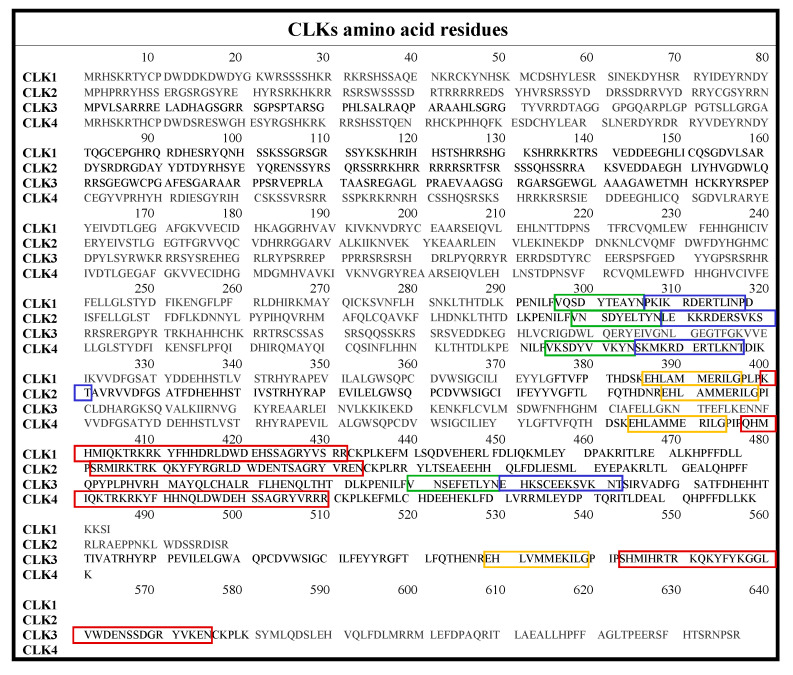
Amino acid sequences of CLK1-4 with color-coded insertions: MAPK-like insertion (red), LAMMER motif (yellow), β-hairpin: β-9 (green), and β-10 (blue) [38].

The CLK family consists of CLK1, CLK2, CLK3, and CLK4, which are briefly summarized in Table 1, with highlighted differences in the ATP-binding region. The full amino acid sequences are given in Figure 2.

**Figure 3:**
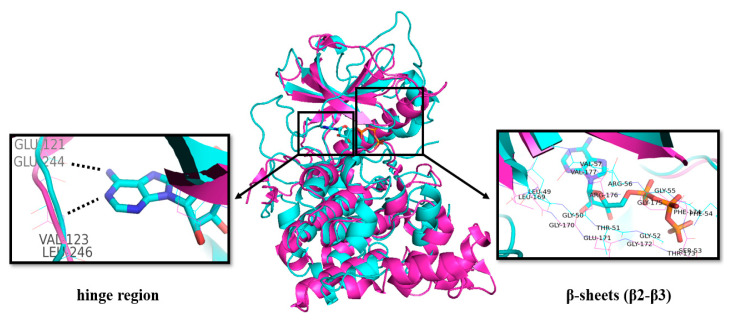
Overlap of the crystal structures of CLK2 (pink, PDB: 3NR9) [43] and PKA with bound ATP (blue, PDB: 1ATP) [44]. Key amino acid residues in the ATP binding region are highlighted by boxes.

The structures of CLKs are quite typical and consist of the N- and C-lobes connected by the “hinge” region of the protein backbone including the ATP binding site, which is conserved as in other kinases [39,40,41]. As for other kinases, the binding of the purine part of ATP is very likely provided by hydrogen bonding to the NH (donor) and CO (acceptor) motifs in the protein backbone (Figure 3). Additional CO is available as an acceptor site [42].

The Glu residue in the back region of the ATP-binding site forms an ion pair with Lys, which facilitates the transfer of the *γ*-phosphate from ATP [40].

The structures of CLKs contain catalytic domains with β-strands and α-helixes distributed between the N- and C-regions. The N-lobe contains three β-strands followed by α-helix and additional two β-strands (Figure 3) [39,45].

**Figure 4:**
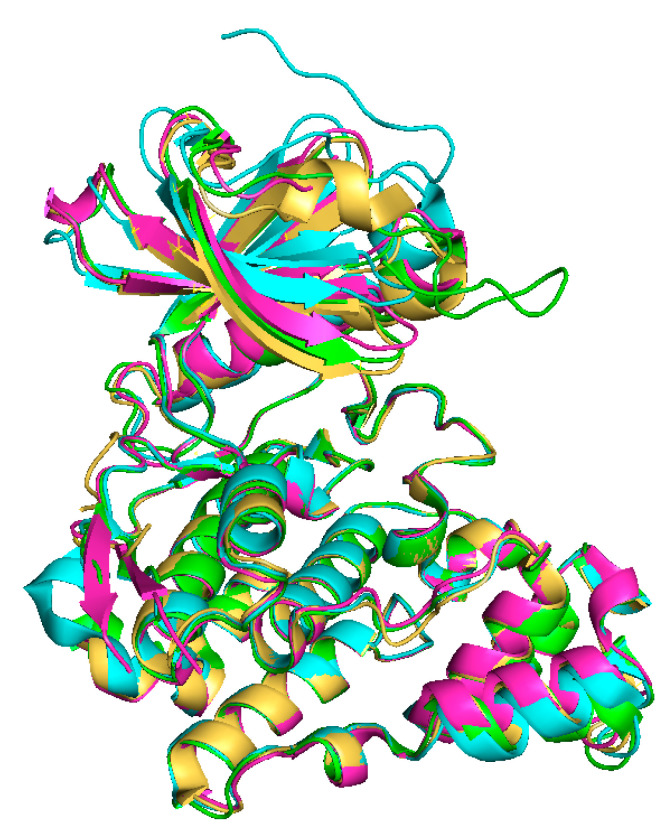
Overlay of X-ray crystal structures: CLK1 (yellow, ligand:12h, PDB: 6I5H) [47], CLK2 (green, PDB: 3NR9) [43], CLK3 (blue, PDB: 6YTW [48], CLK4 (pink, ligand: CX4945, PDB: 6FYV) [45,49].

The tertiary structures of all CLKs are quite similar (Figure 4), nevertheless the structure of CLK3 contains some additional motifs (Figure 2), as described below [39,45,46].

**Figure 5:**
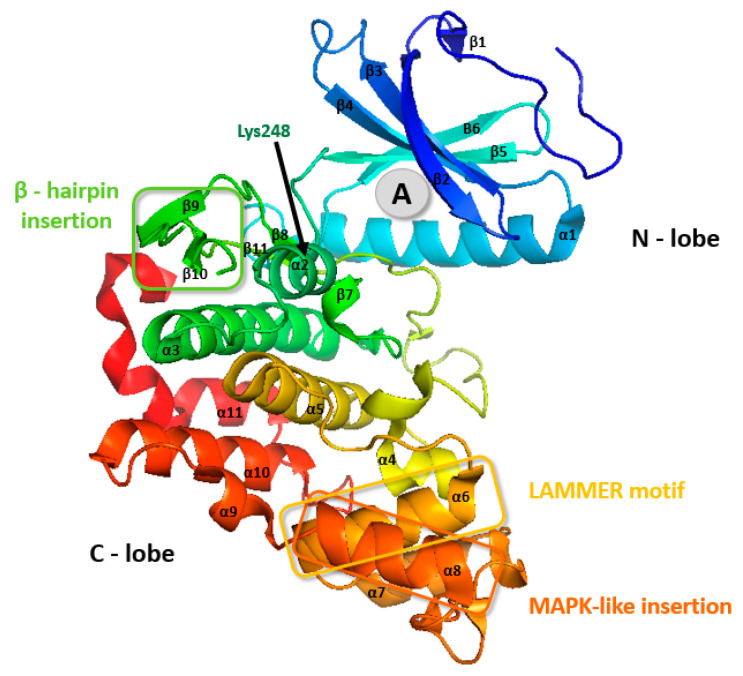
CLK3 crystal structure. The grey circle (A) represents the most common binding pocket for small-molecule inhibitors. PDB: 2EU9 [51].

CLK3 contains a small insert between stands β6 and β9 in the kinase core that interacts with a hydrophobic pocket near the hinge region that connects the N- and C-lobes [50]. The C-lobe of CLK3 is formed by three conserved regions: the LAMMER motif, mitogen-activated insertion, the MAPK-like insertion containing an α8 (residues 419–427), and β-hairpin insertion (Figure 2 and Figure 5) [45,50].

There are two “extra” elements that are not present in CLK1: the first one consists of inserted residues in the hairpin (residues 136–145) folding above the N-terminal domain, the second one is a supplementary helical segment at the C terminus (residues 476–481) [39].

Also, CLK3 possesses one Lys248 residue in the entrance of the active site pocket (Figure 5), which modifies the electrostatic surface charge distribution therein—this might be unfavorable for the entry of some CLK inhibitors [45,46]. 

Given the structural features of CLK3 described above, it is perhaps not surprising that various small-molecule inhibitors frequently exhibit different affinities toward CLK3 compared to the other CLKs (see the chapter “Small-molecule CLK inhibitors” below).

## 3. Biology of CLKs

CLKs are evolutionary conserved dual-specificity kinases that are able to phosphorylate serine, threonine, and tyrosine residues [36,52]. CLKs catalyze the phosphorylation of SR proteins, serine, and arginine-rich splicing factors 1-12 (SRSF1-12), which regulate the spliceosome molecular machinery [53,54,55]. SR proteins seem to have very distinct functions based on their phosphorylation state [56], as reported, for example, for SRSF10 (SRp38) [57,58]. Upon hyper-phosphorylation, SR proteins bind to pre-mRNA and help to stabilize interactions between spliceosome components, which results in spliceosome assembly [27,59,60,61]. Conversely, de-phosphorylation of SR proteins by phosphatases is required in the second step for the pre-mRNA splicing [59,60,61] and subsequently for the export of spliced mRNA to the cytoplasm [26,62]. Via this mechanism, CLKs regulate pre-mRNA splicing and consequently also the translation of genetic information into protein structure [63,64].

All CLK isoforms have been found in most tissues and cell types (e.g., prostate, leukocytes, testes, muscle, brain, liver, lung, kidney, thyroid), but their expression levels differ in some tissues. For instance, CLK1 expression level in the testes was significantly lower than those of CLK2-4 [53,65,66,67]. All CLK isoforms are mostly localized in the nucleus; however, in the testes, CLK3 was found to be localized mostly in stress granules in the cytoplasm [16,52,68]. In contrast, SRPKs were found mostly in the cytoplasm and only a minor fraction was observed in the nucleus [69,70].

Interestingly, CLK1/4 activity is highly responsive to physiological temperature changes, which is caused by reversible temperature-dependent rearrangements in the kinase activation segment, reflected in temperature-dependent SR protein phosphorylation, splicing, and gene expression [71]. Specifically, it has been shown that lower body temperature causes activation of CLK1/4, which leads to increased SR protein phosphorylation.

The mRNAs of all CLK isoforms are alternatively spliced. The spliced variants form heterodimers with full-length CLKs, showing that alternative splicing mediated by CLKs is auto-regulated [69]. CLKs are mostly localized in the nucleus, but their localization has been described also in the cytoplasm (e.g., in stress granules) [16,52,68], although less abundantly than SRPKs [69,70].

CLKs are capable of auto-phosphorylation on Tyr residues, while their substrates are phosphorylated on Ser/Thr residues [53]. In contrast to SRPKs, which phosphorylate only Arg-Ser dipeptides, CLKs can phosphorylate both Arg-Ser and Ser-Pro dipeptides—common motifs in all SR proteins [50]. It has been reported that CLK1/2/4 isoforms phosphorylate Aurora B–S331 in the midbody during late cytokinesis, which leads to Aurora B activation and consequently to the regulation of abscission checkpoint [72].

Via regulation of splicing, CLKs (indirectly) affect a variety of biological processes [73,74,75]. For instance, it has been described that CLKs modulate alternative splicing of key Wnt-related genes and consequently the Wnt signaling pathway [76]. CLKs regulate also the splicing of the TAU protein which plays an essential role in the stabilization of microtubules and thereby impacts processes such as cell division and neuronal activity [77,78,79].

The CLK family consists of four isoforms (CLK1-4); however, the knowledge about their individual biological roles is still rather limited.

### 3.1. CLK1

**Figure 6:**
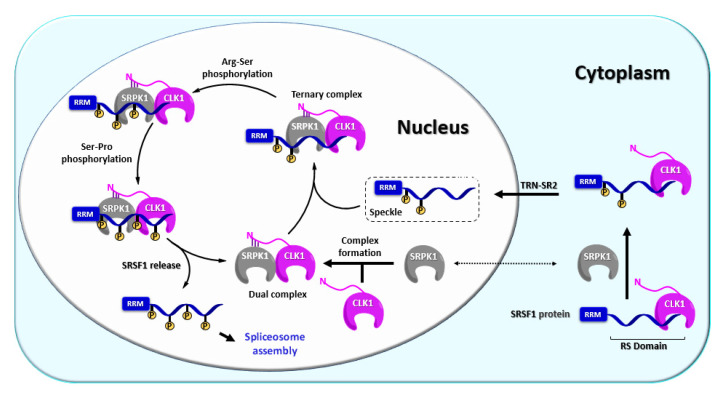
Schematic representation of the phosphorylation mechanism involving CLK1 [39,49,55,68,82,83,84,85].

CLK1 (alternatively referred to as STY) represents one of the first kinases with dual specificity described in the literature and also the most explored CLK isoform up today [36]. CLK1 has been found to regulate alternative splicing of its own gene *clk1* via exon 4 skipping and intron 4 retention [80]. Thus, CLK1 forms an auto-regulatory loop where the catalytically active CLK1 triggers the expression of truncated isoforms CLK1^T1^ and CLK^T2^ [80]. In contrast, extrinsic stress factors or CLK1 inhibition promote the expression of full-length CLK1 [80]. CLK1 possesses a diffuse nuclear localization sequence (NLS) in the N terminus that is responsible for the formation of oligomeric CLK1, which is likely unable to pass through nuclear pores [81]. On the other hand, NLS strongly interacts with its substrate SRSF1 (SR protein), which is very likely important for the nuclear import of CLK1 via a “piggyback” mechanism, where CLK1 is transported inside the nucleus together with the TRN-SR2/SRSF1 complex (Figure 6). This mechanism has been supported by various experiments, for example, disruption of SRSF1 protein transport by TRN-SR2 knockdown or mutation of the SRSF1 NLS impaired CLK1 nuclear localization [68].

Not only nuclear import but also nuclear function has been described most thoroughly for the CLK1 isoform, namely its regulation mechanism of the SR protein SRSF1 [55,68,85,86]. In this process, CLK1 and SRPK1 work co-operatively as a complex [55]. The complex containing CLK1 in active form first recruits hypo-phosphorylated SRSF1 from nuclear speckles (also termed interchromatin granule clusters), which act as a pool of SR proteins in the nucleus, whereby a ternary complex CLK1-SRPK1-SRSF1 is formed (Figure 6). The ternary complex executes full phosphorylation of SRSF1 and subsequently releases it (Figure 6) [55,84,85]. Alternatively, CLK1 itself can form a complex with SRSF1 and catalyze full phosphorylation first. Subsequently, SRPK1 can engage to create the ternary complex CLK1-SRPK1-SRSF1 [85]. The interaction between CLK1 N-terminus and SRPK1 kinase domain holds the complex CLK1-SRPK1 together but also facilitates the release of hyper-phosphorylated SRSF1 from the ternary complex, which is a prerequisite for the assembly of spliceosome [55]. It has been also suggested that the interaction between CLK1 and SRPK1 anchors the SRPK1 in the nucleus, thereby increasing SRPK1 concentration in the nucleoplasm [55]. In addition, CLK1 phosphorylates the splicing factor SPF45 (non-SR protein) on eight serine residues, regulating cell migration and invasion (SPF45 overexpression promotes both processes) [87]. 

CLK1 can be phosphorylated by AKT2, which promotes CLK1-mediated SR protein phosphorylation [88]. In addition, CLK1 also activates KKT2 via phosphorylation at the S508 residue, which is crucial for kinetochore assembly [89], documenting that the CLK1 is a component of a wider signaling network.

In addition, CLK1 can regulate autophagy—CLK1 inhibition or knockout induces autophagy via activation of the mTOR/PI3K pathway [90,91].

These recent findings suggest it may be desirable to evaluate the effect of CLK inhibitors on splicing in the context of the SRPK1-CLK1 complex, rather than using the isolated CLK1 kinase [85]. 

### 3.2. CLK2

Specifically for CLK2, it has been found that it can be stabilized by AKT in response to feeding and acts as a suppressor of the peroxisome proliferator-activated receptor γ coactivator (PGC-1*α*), thus modulating fatty acid oxidation and ketogenesis [92]. In addition, it has been reported that CLK2 phosphorylates the protein phosphatase 2A (PP2A), which leads to assembly of the heterotrimeric PP2A holoenzyme, subsequent AKT dephosphorylation, and attenuation of AKT activity [93]. CLK2 phosphorylation by activated AKT is an important regulatory mechanism promoting cell survival following ionizing radiation [94].

Besides the role of CLK2 in SR protein phosphorylation and splicing regulation, it has been documented that CLK2 and CLK1 also phosphorylate the protein-tyrosine phosphatase PTP-1B, whereby increasing its activity [95].

Recent findings show that CLK2 hyper-phosphorylates Prostate-Associated Gene 4 (PAGE) at multiple Ser- and Tyr-residues. Interestingly, it has been documented that HIPK1-phosphorylated PAGE4 potentiates c-JUN whereas CLK2-phosphorylated PAGE4 diminishes c-JUN activity [96].

### 3.3. CLK3

High levels of CLK3 were detected in mature spermatozoa in mouse testes. Interestingly, CLK3 in these cells is mainly located in the cytoplasm [97].

### 3.4. CLK4

CLK4 and CLK1 are almost identical in amino acid sequence. CLK1/4 proteins supplied through stress-induced splicing of the intron-retaining pre-mRNAs have been found to be essential for the rapid recovery of the phosphorylation state of SR proteins and contribute to the restart of splicing reactions after stress removal. They may thus serve as a guardian maintaining the phosphorylation state of SR proteins to improve the survival of cells exposed to stress [98]. 

## 4. Effects of Altered CLK Expression

On the molecular level, an increased level of CLKs has been found to lead to the following.

Overexpression of active CLKs causes the redistribution of SR proteins within the nucleus and dissolution of speckles [54,56,99], dispersion of eIF4E nuclear speckles [100], complete redistribution of interchromatin granule clusters [101], and specific targeting of SRp55 for degradation by proteasome [102].

The overexpression of CLK1 also affects splicing site selection of pre-mRNA of both its own transcript and adenovirus E1A transcripts in vivo [103]. CLK1 regulates expression of the alternative splicing factor 45 (SPF45), which is frequently overexpressed in cancer. Expression of CLK1 enhances SPF45-induced exon 6 exclusion from Fas mRNA, whereas CLK1 inhibition reduces it [87]. In human stem cells, overexpression of CLK2 leads to the dispersal of speckles and a significant drop in the number of genes in shared neighborhoods [104]. On the morphological and phenotypical levels, altered expression of CLK has been found to elicit different changes, summarized below.

Expression of CLKs in PC12 cells mimic nerve growth factor (NGF)-dependent events, including morphological differentiation and elaboration of neurites up-regulated during HMBA-induced erythroleukemia cell differentiation [105]. Rather conversely, a reduction in transcript levels for CLK1 (and other genes encoding proteins involved in neurite outgrowth, namely NGF, M6a, and GNAQ) was observed in chronically stressed mice [106]. In Xenopus embryos, overexpression of CLK2 induces expression of both anterior and posterior neural marker genes, thereby promoting early neural development [107]. Overexpression of CLK2 significantly increases the growth of HeLa cells and inhibits radiation-induced cell death [94]. CLK2 also acts as an oncogene in breast cancer—it is amplified and overexpressed in a significant fraction of breast tumors and its downregulation inhibits breast cancer growth in cell culture as well as in xenograft models [108]. Recently, CLK3 was found to be markedly upregulated in hepatocellular carcinoma tissues and its expression levels were closely associated with TNM stages and prognosis [109].

Overexpression of CLK2 in the mediobasal thalamus in mice on a high-fat diet can partially reverse the obese phenotype [110]. CLK2 regulates CREB via the attenuation of CREB inhibitory dephosphorylation by PP2Am, which affects diet-induced thermogenesis in brown adipose tissue via increased CREB-dependent expression of UCP1 [93]. There are indications that BAT CLK2 increases diet-mediated energy expenditure and protects against obesity [93]. Moreover, it has been shown that CLK2 is an insulin-regulated suppressor of hepatic gluconeogenesis [111].

## 5. CLKs as Therapeutic Targets

Compromised accuracy of alternative splicing can have a profound impact on human pathogenesis, in particular on tumor development and progression [112]. Recent reports described recurrent change-of-function mutations in splicing factors in a variety of cancers and suggested that abnormal splicing can trigger tumor growth [113,114,115]. As briefly illustrated in the previous chapter and described in greater detail here, there has been increasing experimental evidence that abnormal function and levels of CLKs are present in different cancers.

Cancer cells have general as well as cancer type-specific and subtype-specific alterations in the splicing process that can have prognostic value and contribute to cancer progression. These splicing alterations are often linked to the occurrence of cancer driver mutations in genes encoding either core components or regulators of the splicing machinery, which can make the cells particularly vulnerable to the pharmacological inhibition of splicing [116,117]. More specifically, there has been emerging evidence that splicing kinases are dysregulated in cancer and play important roles in both tumorigenesis as well as therapeutic response to radiation and chemotherapy [16].

A recent report demonstrated that MYC activation, which altered pre-mRNA splicing without the transcriptional regulation of CLKs, can render cancer cells vulnerable to CLK inhibitors with resulting synergistic cell death [118]. The potential therapeutic benefit of CLK inhibition was also demonstrated in vivo in an allograft model of spontaneous, MYC-driven breast cancer [118]. 

Numerous investigations of the cancer-related biology of CLKs have been supported by the use of small-molecule inhibitors (described in the following chapter) with varying degrees of selectivity. Of those, the inhibitor SM08502 recently entered clinical trials for the treatment of advanced solid tumors [76], and the compound CX-4945/silmitasertib (which inhibits CLK2 as well as CK2) [119] for the treatment of cholangiocarcinoma, medulloblastoma, basal cell carcinoma, and multiple myeloma [120]. CX-4945 could be also applicable in the treatment of glioblastoma where CLK2 modulates phosphorylation of Forkhead box O3a (FOXO3a)/p27 and, consequently, cell cycle and survival of tumor cells [121].

As exemplified below, CLKs could be attractive targets in other therapeutic areas beyond oncology. Despite their high homology, overexpression of CLK1 vs. CLK2 was found to have opposite effects on the replication of the HIV-1 virus, while changing the levels of CLK3 and CLK4 had no significant effect [122].

As described previously, the CLK-mediated phosphorylation process is involved in RNA splicing; however, aberrant splicing involving the microtubule-associated protein tau is associated with neurodegenerative diseases such as frontotemporal dementia and Parkinson’s disease and other tauopathies [78,123,124]. Recent reports indicate that CLKs (and other kinases) can be involved in the molecular mechanism of Alzheimer’s disease and [123,125,126] the CLK/DYRK inhibitor Leucettine 41 can activate the autophagic mTOR/PI3K pathway, which may represent a therapeutic option [91]. 

Pharmacological inhibition of CLK1 has been investigated pre-clinically (in mice) for the treatment of Duchenne muscular dystrophy—it resulted in enhanced mutated exon 31 skipping, which led to the production of functional exon 31 skipped dystrophin in cells derived from Duchenne muscular dystrophy patients [127].

CLK1 inhibition and CLK1 knockout effect also splicing of the viral RNA transcript. Specifically, it has been shown that CLK1 knockdown or inhibition reduced influenza A/WSN/33 virus replication in A549 cells [128]. Moreover, CLK1−/− mice infected with influenza A/England/195/2009 (H1N1pdm09) virus had lower levels of virus replication in comparison to wild-type mice, and CLK1 inhibition had a similar effect [128].

CLK2 is also considered as an insulin-regulated suppressor of hepatic gluconeogenesis, contributing to hyperglycemia in diabetes. The mechanism is principally based on direct phosphorylation of the SR domain of the transcriptional coactivator (PGC-1α) by CLK2. This phosphorylation process causes repression of gene expression in the gluconeogenesis, resulting in hypoglycemia [111]. Along this line, it has been suggested that the levels of insulin could be regulated by CLK2 inhibition [88,111].

In neurology, inhibition of CLK2 was reported to restore normal sociability of Shank3-deficient mice (likely via activation of AKT activation) and thus could be therapeutically relevant for the treatment of the autistic spectra disorder Phelan-McDermid syndrome (PMDS) [129].

Finally, it has been reported that inhibition of CLK3 in *Plasmodium falciparum* (*Pf*CLK3) represents a promising approach for the treatment of malaria via preventing the splicing of essential parasite genes. It has been demonstrated that CLK3 inhibition can kill multiple species of malaria parasites at the blood stage and at the liver-stage and block transmission of the parasite to mosquitoes [130,131]. Another indication where CLK inhibition could be therapeutically relevant is Legionellosis, where CLK inhibition (by TG003) was demonstrated to reduce Legionella growth within mouse macrophages [132].

## 6. Small-Molecule CLK Inhibitors

Over the last two decades, structurally diverse CLK inhibitors have been described in the literature, with the majority of them being published in recent years. This chapter provides a chronological summary of individual compounds and/or selected representatives of individual structural series, with the focus on substances that have been most thoroughly profiled and could thus serve as chemical probes for chemical biology studies (Table 2). For better comparison, Table 2 also contains data for structurally similar DYRKs and HIPKs, since achieving selectivity against those two sub-families is not trivial and represents a significant challenge in the development of selective CLK inhibitors. Additional typical off-targets of many CLK inhibitors are PIM and/or CK1 kinases. 

Of note, most of the reported CLK inhibitors interact relatively weakly with CLK3—only 4 out of 24 compounds exhibit IC_50_/K_d_ values towards CLK3 < 100 nM. Table 2 contains the published values obtained in primary biochemical assays; however, for their direct comparison it is important to keep in mind that these data were often obtained from various sources/assays or from the same (similar) assay but under unequal conditions (e.g., using different concentrations of ATP). On the other hand, some studies report a direct comparison of several (usually two or three) inhibitors from different classes/publications, profiling them in the same assay [76,133,134,135,136].

An additional brief summary is given for each of the inhibitors, with the main focus on their selectivity and (therefore) potential to be used as quality CLK chemical probes in chemical biology. It is likely that in the near future, highly selective tool compounds will help further unravel the complexity of CLK biology and validate the therapeutic potential of CLK inhibition. Of the listed CLK inhibitors, we highlight those that (in our opinion) fulfill the criteria for a quality probe. However, it is highly recommended to confirm the context of the obtained biological data in original reports prior to use and (whenever possible) use at least two chemically different quality chemical biology probes to minimize the risk of compound-specific off-target effects.

Some of the compounds exhibiting sufficient activity in the cell and in vivo have been pre-clinically profiled as anti-tumor agents [76,135,137]. Of note, the therapeutic potential of CLK inhibitors might be enhanced by simultaneous inhibition of additional targets [136,137].

**Table 2:** In vitro activities of known CLK inhibitors, including their typical off-targets. The IC_50_ values (in nM) are given as numbers, Kd values (in nM) are specified. In cases where IC_50_/K_d_ is not available, the compound’s potency is expressed as a percentage of the enzyme’s residual activity at the concentration of the inhibitor specified in brackets. CLK probes recommended by SGC are highlighted in green. ND: no data found.

	CLKs				Structurally Similar Kinases						Other Significant Targets	Kinases Screened
Compound	CLK1	CLK2	CLK3	CLK4	DYRK1A	DYRK1B	DYRK2	HIPK1	HIPK2	HIPK3		
**TG003** [64,134,138]	19 (K_d_)	95 (K_d_)	inactive	30 (K_d_)	12 (K_d_)	130 (K_d_)	ND	ND	ND	ND	CK1*δ* = 150 CK1ε = 300 CK1γ2 = 270 CK1γ3 = 290 PIM1 = 130 PIM3 = 280 YSK4 = 290 (K_d_)	~402 kinases
**ML106** [134,138]	37 (K_d_)	680 (K_d_)	470 (K_d_)	50 (K_d_)	27 (K_d_)	430 (K_d_)	ND	ND	ND	ND	EGFR = 230 (K_d_)	402 kinases
**ML167** [138]	1522	1648	inactive	136	inactive	4420	ND	ND	ND	ND	Not published	442 kinases
**CX-4945** [133,139,140]	3.3	2.9	67	23	14	ND	5% (0.5 µM)	11% (0.5 µM)	15% (0.5 µM)	7% (0.5 µM)	CK2α = 1.5 PIM1 = 216 TBK1 DAPK2 ZIPK FLT1	~235 kinases
**KH-CB19** [141]	20	ND	530	ND	55	ND	ND	ND	ND	ND	PIM1/3 SGK085	106 kinases (thermal shift assay)
**Leucettine L_41_** [142]	71	720	inactive	64	60	44	73	1% (10 µM)	11% (10 µM)	4% (10 µM)	GSK3α = 210 PIM1 IRAK1 TAOK1	402 kinases
**ML315** [143]	68	231	inactive	68	282	1156	ND	ND	ND	ND	PRKCE MAP3K1 CK1ε	442 kinases
**Thiophene 48** [144]	110	22% (5 µM)	69% (5 µM)	1% (5 µM)	100	70	40	91% (5 µM)	ND	ND	-	102 kinases
**3A5** [145]	51	68	346	ND	260	ND	61% (1 µM)	inactive	inactive	inactive	CK1γ2	~140 kinases
**Cpd-2** [146]	1.1	2.4	ND	ND	ND	ND	ND	ND	ND	ND	SRPK1 = 200 SRPK2 = 310 SRPK3 = 230	28 kinases
**SRI-29329** [147]	78	16	>1000	86	95% (1 µM)	ND	ND	ND	ND	ND	-	29 kinases
**T3** [135,148]	0.67	15	110	ND	260	230	ND	inactive	inactive	ND	-	71 kinases
**Compound 25** [90]	2	31	inactive	8	138	690	inactive	inactive	inactive	inactive	-	368 kinases
**TG693** [127]	113	85% (1 µM)	88% (1 µM)	ND	16% (1 µM)	ND	23% (1 µM)	inactive	ND	inactive	HASPIN	~313 kinases
**Indazole1** [133]	12	10	2250	12	73	ND	ND	ND	ND	ND	-	34 kinases
**KuWal151** [149]	88	510	inactive	28	inactive	inactive	inactive	ND	ND	ND	-	IC_50_ for 14 kinases
**T-025** [118]	4.8 (K_d_)	0.096 (K_d_)	6.5 (K_d_)	0.61 (K_d_)	0.074 (K_d_)	1.5 (K_d_)	32 (K_d_)	55 (K_d_)	96 (K_d_)	5% (0.3 µM)	HIPK4 YSK4 IRAK4 FLT ERK8	468 kinases
**CC-671** [137]	300	6.3	60% (3 µM)	ND	104	157	ND	97% (3 µM)	96% (3 µM)	92% (3 µM)	TTK = 5 DYRK3 = 99 PhKγ1 = 136 TSSK1 = 452	225 kinases
**Pyrido** **[3,4-*g*]quinazoline 9m** [150]	18	ND	ND	ND	39% (1 µM)	ND	ND	ND	ND	ND	CDK5 CK1 GSK3	-
**SGC-CLK-1** [151]	13	4	363	46	ND	ND	ND	50	42	ND	ERK8 NEK7 PIP5K2B STK16	403 kinases
**SM08502** [76]	8	1	22	1	1	1	3	ND	23	21	additional 14 kinases with IC_50_ < 50 nM	466 kinases
**MU1210** [47,152]	8	20	Inactive	12	213	956	1309	187	29	159	GSK3α PIM1/1 HASPIN	210 kinases
**AB1** [89]	ND	ND	ND	ND	ND	ND	ND	ND	ND	ND	BTK = 2 hERGFR = 2	ND
**TCMDC-135051** [130,131]	ND	17% (1 µM)	40	ND	71% (1 µM)	ND	99% (1 µM)	83% (1 µM)	56% (1 µM)	93% (1 µM)	MNK1 MAP4K3 CAMKKb CDK9 IRAK1 TGFBR1 PHK	140 kinases

**Figure 7:**
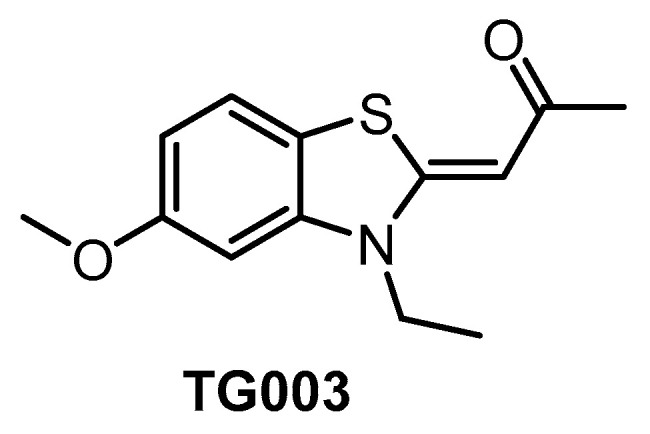
Structure of the compound TG003.

TG003 (Figure 7) represents one of the first CLK inhibitors (published in 2004) [64] and its activities in biochemical assays have been determined several times with slightly different results [64,134,153]. TG003 is active also in cell-based assays and in vivo. In the cell, the inhibitor can elicit suppression of the exon skipping and serine/arginine-rich protein phosphorylation, dissociation of nuclear speckles, and Clk1/Sty-dependent alternative splicing [64]. A broader screening against 402 kinases revealed significant off-target activities, indicating that the use of TG003 as a chemical probe is likely problematic [134]. Despite this fact, the compound is still being widely used in life sciences as a CLK inhibitor.

**Figure 8:**
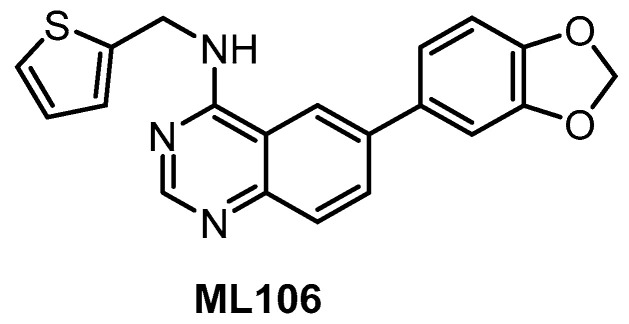
Structure of the compound ML106.

The quinazoline-based inhibitor ML106 (Figure 8) was published in 2009 [134]. ML106 (analog of ML167) inhibits CLK1/2/3/4 with K_d_ values of 37 nM, 680 nM, 470 nM, and 50 nM, respectively. ML106 was profiled in a panel of 402 kinases; detailed results of the profiling are not available, but significant off-targets are DYRK1A, DYRK1B, and EGFR with K_d_ values 27 nM, 430 nM, and 230 nM, respectively [134]. Of note, the published IC_50_ values (CLK1 IC_50_ = 59 nM, CLK2 IC_50_ = 1902 nM, CLK3 IC_50_ = 6936 nM, CLK4 IC_50_ = 39 nM) do not correlate well with the K_d_ values above [153]. Other quinazoline-based analogs were also profiled in panels of 402 or 442 kinases, which revealed their very good selectivity [153]. Some of them were further tested in the Caco-2 permeability assay or for cellular efflux, suggesting that selected analogs might be used as tool compounds for cell-based experiments; however, no cellular activities have been published thus far [153].

**Figure 9:**
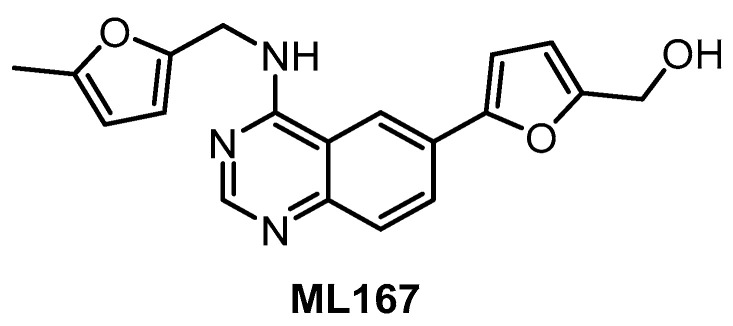
Structure of the compound ML167.

To our best knowledge, the compound ML167 (Figure 9) represents the most selective CLK4 inhibitor (IC_50_ = 136 nM), according to the data obtained in biochemical screening (Table 2) [153]. Although ML167 is not the most potent CLK inhibitor, it is selective with respect to other family members CLK1/2/3 and (importantly) DYRK1A/B (Table 2). The selectivity was determined by testing in a panel of 442 kinases [153]; unfortunately, neither the results of this kinome-wide screening nor information about additional off-targets are available. Likewise, there are no data on the compound’s activity in the cell. Therefore, any utilization of ML167 as a chemical probe for CLK4 should be considered with caution. Many ML167 analogs with different CLK and DYRK selectivity profiles have been synthesized; more detailed information can be found in the original report [153].

**Figure 10:**
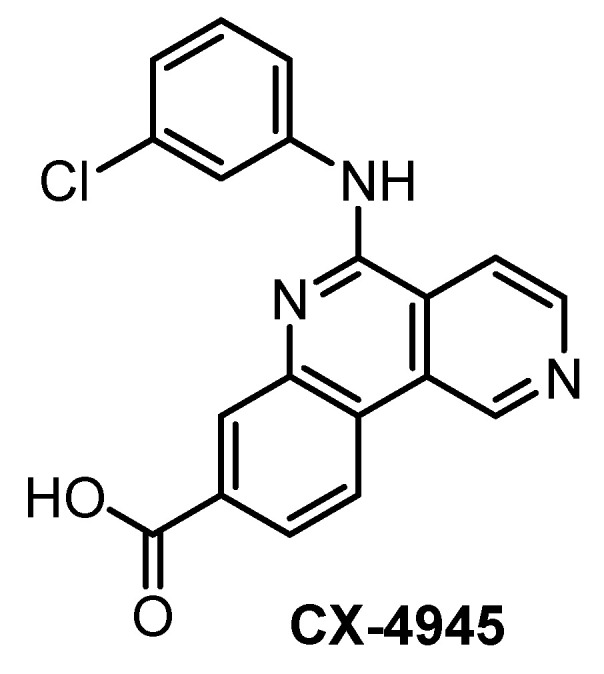
Structure of the compound CX-4945.

CX-4945 (Figure 10) has been primarily reported, marketed, and used as a highly selective and potent inhibitor of CK2 since 2011 [140]. However, CX-4945 strongly inhibits also CLKs and DYRK1A [133]. In addition, it interacts significantly with other kinases such as HIPKs or PIM kinases [133,139].

**Figure 11:**
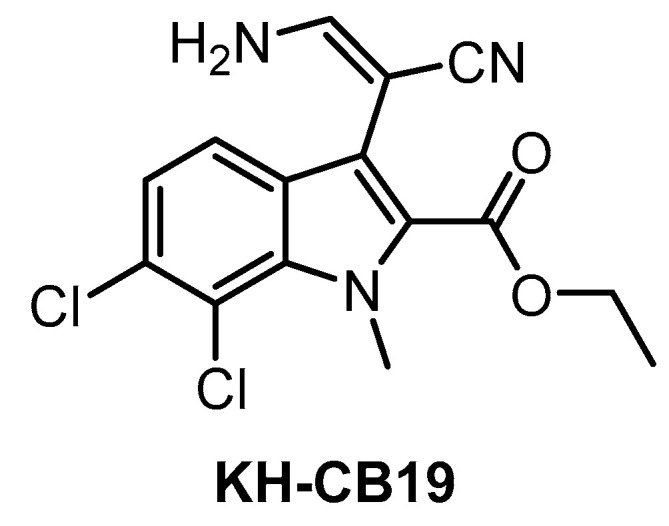
Structure of the compound KH-CB19.

KH-CB19 (Figure 11), published in 2011, was among the first CLK inhibitors found to be superior to TG003 [141]. Based on IC_50_ values obtained in biochemical assays, the compound is a potent inhibitor of CLK1 and DYRK1A. The selectivity profile, evaluated by thermal shift assay against 106 kinases, indicates also strong inhibition of CLK4 and some off-target activity against PIM1/3 and SGK085 [141]. KH-CB19 also exhibits CLK inhibitory activity in the cell [141]. Utilization of KH-CB19 as a chemical probe should be done with caution and ideally after further profiling across the kinome. The utilization in vivo might be problematic since the molecule contains potentially labile ester functionality.

**Figure 12:**
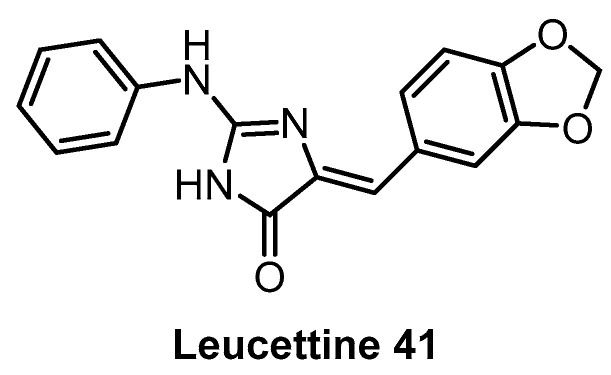
Structure of Leucettine 41.

Leucettine 41 (Figure 12) was developed by modification of the natural product Leucettamine B [142]. The compound strongly inhibits CLK1/4 and DYRK1A/B [142]. However, the data for DYRK2 are rather inconsistent (IC_50_ = 73 nM; Kd = 450 nM), since for a competitive inhibitor, K_d_ is typically smaller than IC_50_ [142]. Leucettine 41 is only weakly active towards CLK2/3 (Table 3). Testing in a panel of 402 kinases at 10 µM concentration revealed a good selectivity profile; additional off-targets being HIPK1/3 and GSK3A [142]. Interestingly, GSK3A inhibition in the cell was not observed while in cell inhibition of DYRK1A was confirmed [142]. Leucettine 41 could be a useful chemical probe, but additional profiling would be desirable (such as determination of the CLK engagement in the cell or in cell activity against HIPKs).

**Figure 13:**
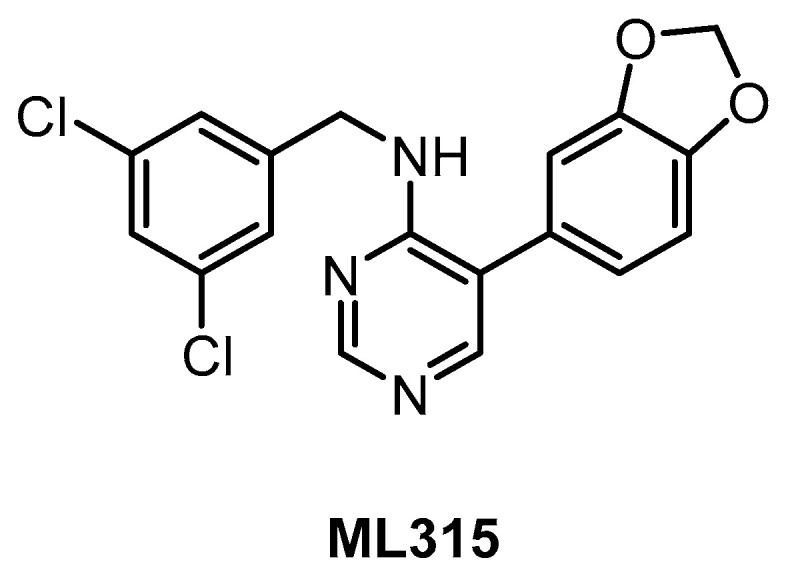
Structure of the compound ML315.

ML315 (Figure 13) was published in 2013 as a selective CLK/DYRK1A inhibitor [143]. The compound inhibits both CLK1/4 with IC_50_ of 68 nM and, somewhat more weakly, CLK2 and DYRK1A (IC_50_ = 231 nM and 282 nM, respectively) [143]. A very good selectivity profile was confirmed by screening in a panel of 442 kinases whereby PRKCE was revealed as the only off-target showing <10% residual activity at 10 µM concentration [143]. Although the microsomal stability of ML315 is low, it has appropriate physicochemical properties for in cell studies and represents a useful chemical probe for CLK/DYRK biology [143].

**Figure 14:**
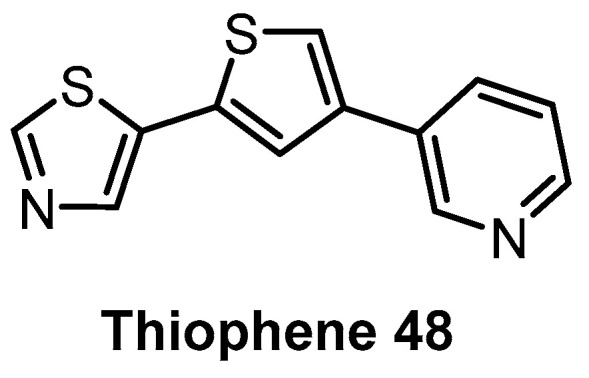
Structure of the compound Thiophene 48.

Based on the natural product Harmine, which is moderately active against CLK1 and DYRK kinases, Thiophene 48 (Figure 14) has been identified as a CLK1/DYRK1/DYRK2 inhibitor [144]. Profiling in a panel of 102 kinases at 5 µM concentration revealed decent selectivity, with strong inhibition of CLK4 [144]. In cell activity has been demonstrated as well [144]. Further profiling would be necessary to determine whether Thiophene 48 fulfills the criteria for a quality chemical probe (for CLK1/4 and DYRKs).

**Figure 15:**
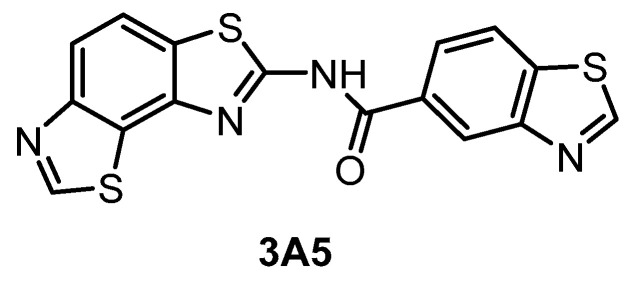
Structure of the compound 3A5.

The compound 3A5 (Figure 15) was reported in 2015 as a CLK inhibitor with a novel benzobisthiazole scaffold [145]. Furthermore, 3A5 has a strong affinity towards CLK1/2 and only moderate activity against CLK3 and DYRK1A. Importantly, it is inactive against structurally similar HIPKs and only weakly active against DYRK2. Unfortunately, the selectivity was determined in a panel of only 142 kinases at 1 µM concentration and the CLK4 and DYRK1B inhibitory activities have not been determined at all. Although 3A5 shows some selectivity for CLKs vs. DYRKs, its utilization as a probe cannot be recommended as its in cell activities have not been demonstrated thus far.

**Figure 16:**
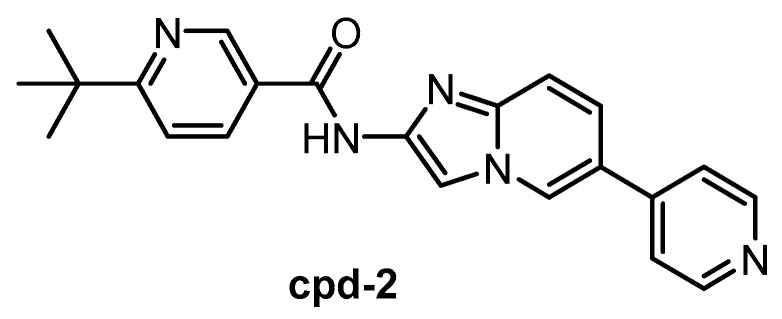
Structure of the compound cpd-2.

The CLK1/2 inhibitor cpd-2 (Figure 16) was published in 2015, together with two analogs cpd-1 and cpd-3 [146]. Cpd-2 possesses high selectivity for CLK1/2 vs. SRPK1/2/3. However, the inhibitors have been screened against only 28 kinases. Therefore, the compound cannot be recommended as a chemical probe at this point.

**Figure 17:**
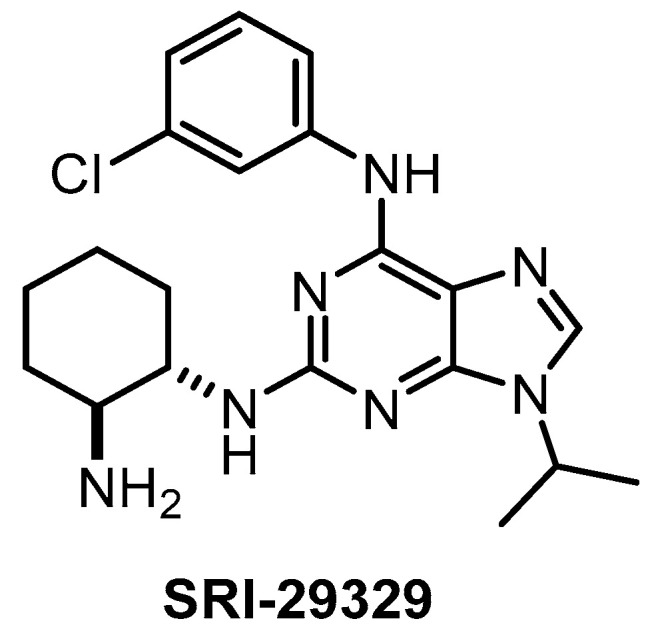
Structure of the compound SRI-29329.

The CLK1/2/4 inhibitor SRI-29329 (Figure 17) was published in 2016, together with its analogs [147]. Some members of the series show dual inhibitory activity towards CLK1/24 and CDK1/4 (such as CGP-74514A, which is incorrectly being sold and used as a selective CDK1 inhibitor) [147]. Of note, SRI-29329 is inactive against DYRK1A [147]. Since SRI-29329 has been profiled against only 29 kinases, it is not recommended to use it as a chemical probe [147]. 

**Figure 18:**
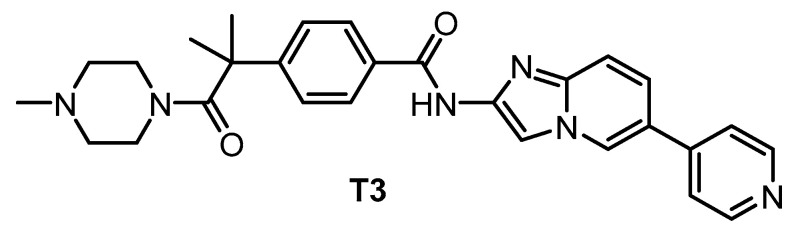
Structure of the compound T3.

The pan-CLK inhibitor T3 (Figure 18), based on cpd-1/2/3, was published in 2017 [135]. The compound is a potent inhibitor of CLK1, CLK2, and CLK3—the IC_50_ values determined in biochemical assays are 0.67 nM, 15 nM, and 110 nM, respectively. T3 exhibits good selectivity against DYRK1A and DYRK1B (IC_50_ = 260 nM and 230 nM, respectively) [135]. Other significant off-targets have not been found thus far. However, the compound has been screened against only 71 kinases at 0.1 µM and 1 µM concentrations [135]. Cellular activity of T3 has been confirmed in several assays; its cellular potency based on NanoBRET assay is 4 nM, 17 nM, and 2 nM towards CLK1/2/4, and 32 nM and 67 nM towards DYRK1A/B, respectively [148]. It has been also shown that T3 induces a decrease in the phosphorylation state of SRSF protein in a time- and dose-dependent manner at 10–1000 nM concentration in HeLa cells [148]. Importantly, the testing was performed in parallel with a negative control compound [148]. As a result, T3 can be recommended as a quality chemical probe for CLK1/2/4, ideally in combination with another selective inhibitor.

**Figure 19:**
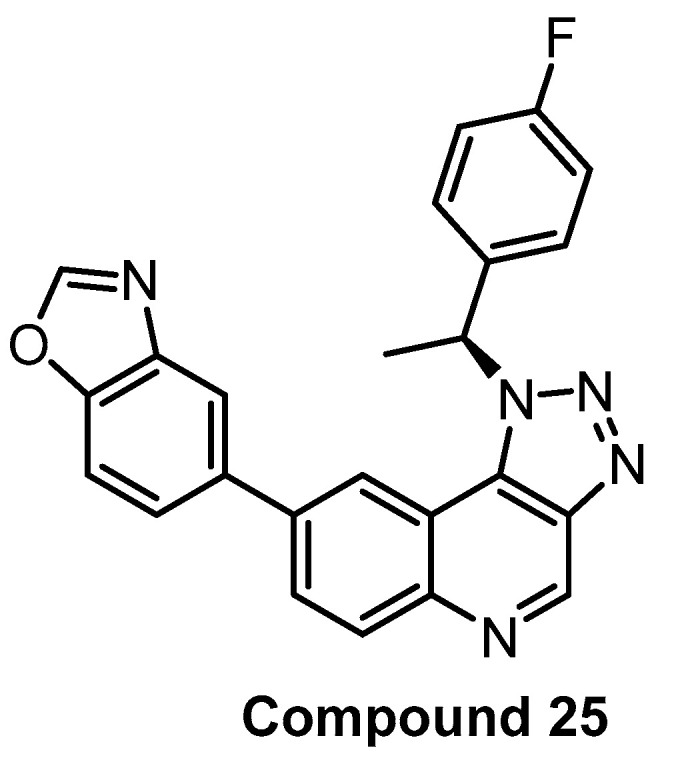
Structure of Compound 25.

In 2017, Compound 25 (Figure 19) was published as a CLK1 inhibitor [90]. While the compound exhibits the highest potency towards CLK1 (IC_50_ = 2 nM), it is also highly active towards CLK2 and CLK4 (IC_50_ = 31 nM and 8 nM, respectively), but practically inactive against CLK3. The most significant off-target outside of the CLK subfamily is DYRK1A (IC_50_ = 138 nM). Compound 25′s high in vitro selectivity was confirmed in the panel of 387 human protein kinases at 10 µM concentration. The cellular activity was investigated via the effect on SR protein phosphorylation. In addition, Compound 25 induced autophagy in BNL CL.2 and SKOV-3 (human ovarian cancer cell line) cell lines. The pharmacokinetic profile (in mice) is also available in the original publication [90]. Unfortunately, an inactive control compound as well as the determination of cellular target engagement are missing. Compound 25 can be considered a useful tool compound for CLK1/2/4, but it would be desirable to supplement it with an inactive control compound.

**Figure 20:**
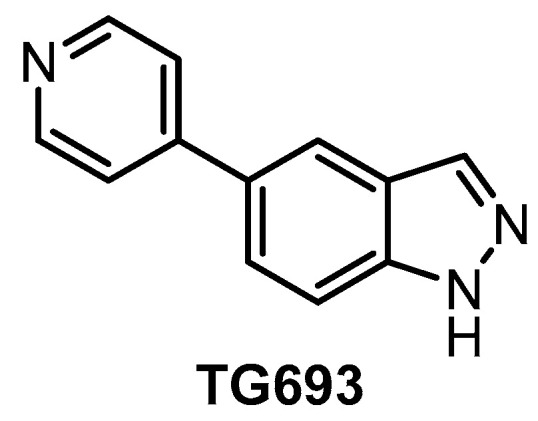
Structure of the compound TG693.

The orally available CLK inhibitor TG693 (Figure 20) was reported in 2017 [127]. The compound shows good selectivity, based on screening against a panel of 313 kinases at 1 µM concentration; however, its in vitro activity towards CLK1 (IC_50_ = 113 nM, biochemical assay) is somewhat lower compared to other known CLK inhibitors. TG693 exhibits only a weak affinity for CLK2/3; and to our knowledge, the activity against CLK4 has not been determined. The most significant off-targets are HASPIN, DYRK1a, and DYRK2, with residual activities of 7%, 16%, and 23%, respectively, upon treatment with 1 µM TG693. Although TG693 does not fulfill the criteria for a quality probe, it still might be one of the best tools for selective inhibition of CLK1. TG693 exhibits activity in the cell and in vivo and its pharmacokinetic profile in mice is superior to that of TG003 [127]. 

**Figure 21:**
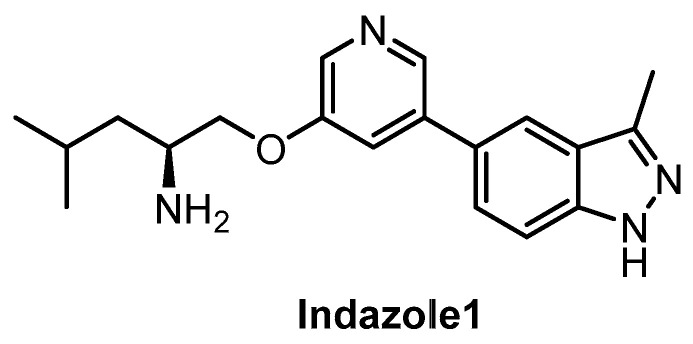
Structure of the compound Indazole1.

Indazole1 (Figure 21) was published recently as an inhibitor of CLKs, active in vivo [133]. The compound potently inhibits CLK1/2/4 and DYRK1A in vitro. However, the data on the selectivity of Indazole1 are not sufficient, since the compound has been profiled against only 34 kinases [133]. Therefore, its use as a chemical probe should be avoided at this point.

**Figure 22:**
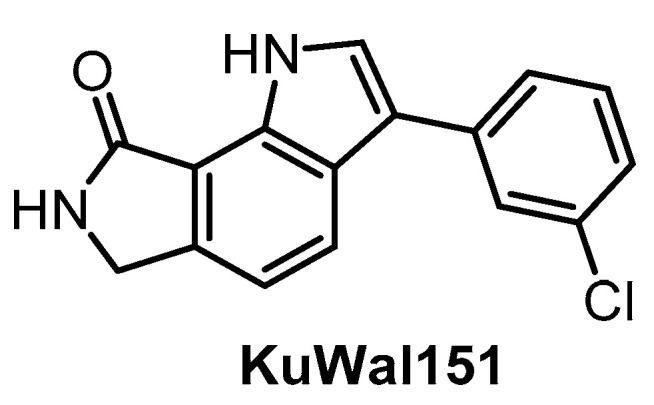
Structure of the compound KuWal151.

The potent CLK inhibitor KuWal151 (Figure 22) was published in 2018 [149]. The determined IC_50_ values for CLK1/2/4 are 88 nM, 510 nM and 28 nM, respectively [149]. Interestingly, KuWal151 is inactive towards DYRK1A/B and DYRK2 [149]. However, the compound’s selectivity profile across the kinome has not been published thus far and only IC_50_ values for 14 kinases have been reported. The antiproliferative activity of KuWal151 was demonstrated in a panel of 57 cancer cell lines—in most cases, the compound was active at sub-micromolar concentrations [149]. 

**Figure 23:**
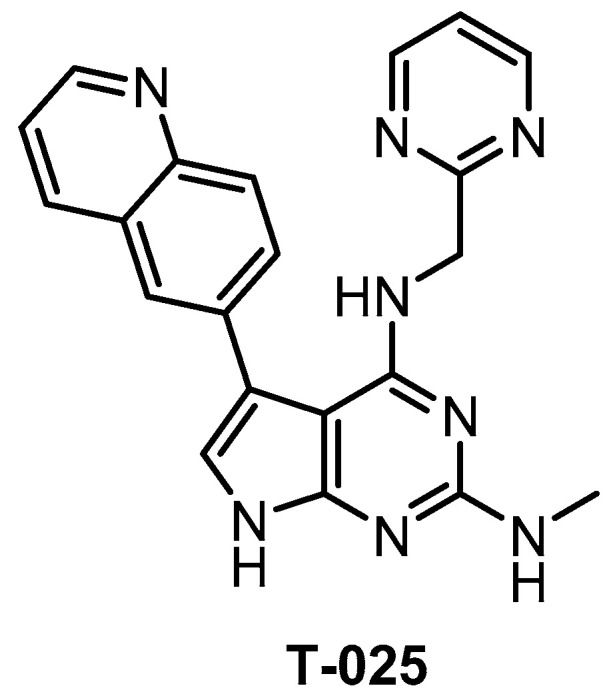
Structure of the compound T-025.

T-025 (Figure 23) was reported in 2018 as a potent pan-CLK inhibitor, which (unlike most CLK inhibitors) exhibits also high potency against CLK3 (K_d_ = 3.5 nM) [118]. The analysis of the crystal structure revealed that the inhibitor interacts with Glu244 and Leu246 in the CLK2 hinge region [118]. The compound’s good selectivity profile was revealed via screening against 468 kinases at 0.3 µM concentration [118]. In comparison to the inhibitor T3, T-025 exhibits lower selectivity towards DYRK1A/B and it inhibits also DYRK2, HIPKs, IRAK4, and YSK4 with K_d_ < 100 nM [118]. In addition, T-025 exhibits cellular and in vivo activity and according to the authors, it represents the first reported CLK inhibitor with anti-tumor efficacy [118]. Unfortunately, the cellular potency and selectivity have not been determined. Although T-025 does not fulfill the criteria for a quality chemical probe for CLKs, still it might be a useful tool compound in some cases, considering its potency against CLK3 and in vivo activity.

**Figure 24:**
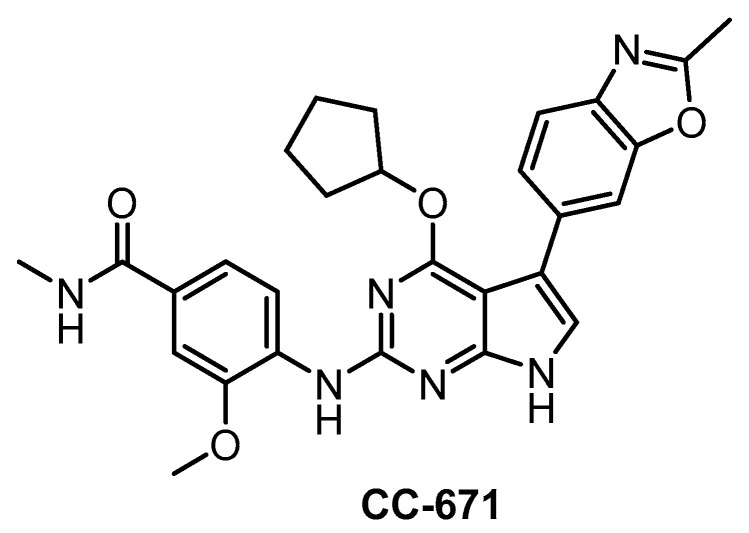
Structure of the compound CC-671.

In 2018, CC-671 (Figure 24) was reported as CLK/TTK inhibitor [137]. Interestingly, this compound exhibits some selectivity for CLK2 (IC_50_ = 6 nM) vs. CLK1 (IC_50_ = 300 nM) and especially CLK3 (60% residual activity at 3 µM concentration), based on biochemical assay [137]. Unfortunately, the compound’s ability to inhibit CLK4 is unknown as well as its selectivity profile in the cell. Although CC-671 does not fulfill the criteria for a quality probe, further profiling (and potentially structural modifications) may potentially lead to an interesting tool compound for specific inhibition of CLK2. In addition, CC-671 has been used in various cell-based and in vivo assays where it elicited growth inhibition and induction of apoptosis in various types of cancer cells [137]. The in vivo efficacy was shown in two cell line-derived and one patient tumor-derived xenograft models of triple-negative breast cancer. This therapeutic effect was credited to dual inhibition of CLK2/TTK [137]. 

**Figure 25:**
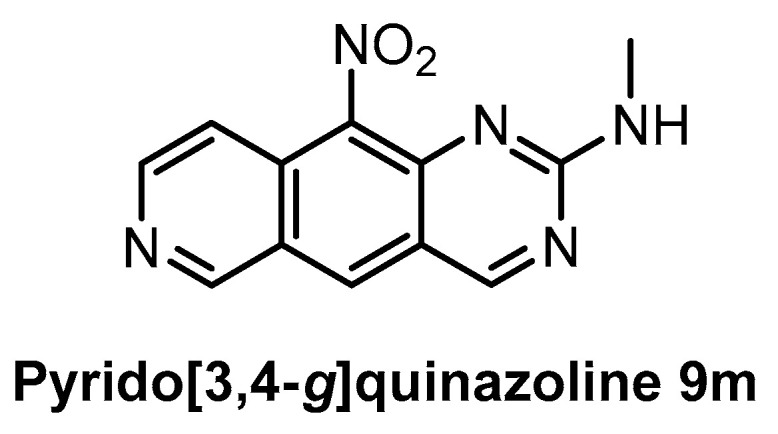
Structure of the compound pyrido[3,4-*g*]quinazoline 9m.

The family of pyrido[3,4-*g*]quinazoline-based compounds inhibiting CLK1, DYRK1A, CDK5, CK1, and GSK3 was published in 2019 [150]. More than 20 different analogs with varying selectivity were described in the report. The most active compound towards CLK1 is the pyrido[3,4-*g*]quinazoline 9m (Figure 25), exhibiting IC_50_ of 18 nM [150]. The inhibitory activities against DYRK1A, CDK5, CK1, and GSK3 were determined at fixed concentrations (10 µM and 1 µM); the residual kinase activities at 1µM concentration are: DYRK1A = 39%, CDK5 = 73%, CK1 = 98%, and GSK3 = 70%. However, more detailed information about the kinome-wide selectivity is not available [150].

**Figure 26:**
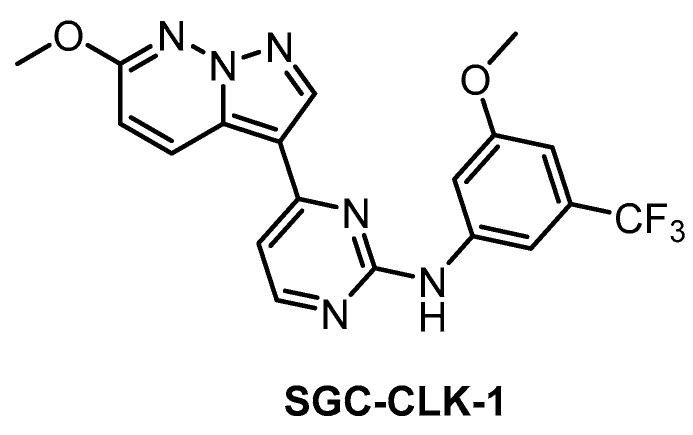
Structure of the compound SGC-CLK-1.

The SGC developed CLK1/2/4 inhibitor SGC-CLK-1 (Figure 26) in collaboration with Luceome Biotechnologies [151]. The compound’s activity towards CLK1/2/4 was determined in biochemical assays and confirmed in the cell by NanoBRET assay (in cell IC_50_ = 165/100/70 nM for CLK1/2/4, respectively) [151]. SGC-CLK-1 was tested in a panel of 403 kinases and the potential off-targets (HIPK1/2, ERK8, NEK7, PIP5K2B, STK16) were then evaluated in NanoBRET assay [151]. As a result, the only off-target found to be partially inhibited in the cell was STK16 [151]. A negative control compound was tested in parallel with SGC-CLK-1. This inhibitor represents an excellent tool compound for cell-based experiments and it can be recommended as a quality probe for CLK1/2/4.

**Figure 27:**
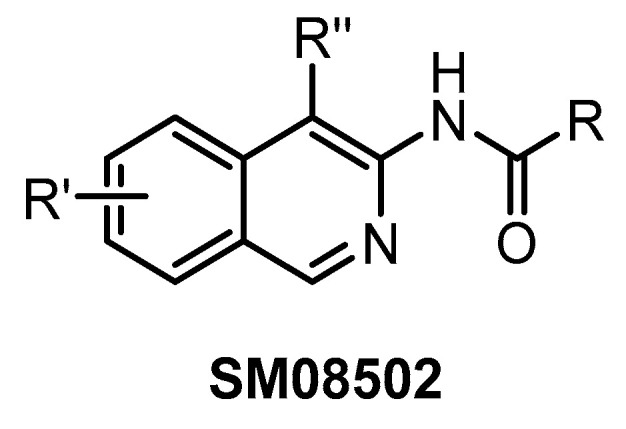
Generic structure of the compound SM08502.

SM08502 (Figure 27) represents the first CLK inhibitor that entered clinical trials [76]. The exact structure of this isoquinoline-based compound has not been disclosed so far. Since the compound inhibits several off-targets (19 additional kinases with IC_50_ < 50 nM), it is not suitable for utilization as a chemical probe. On the other hand, its favorable pharmacological properties and anti-tumor efficacy enabled the initiation of Phase I of clinical trials using SM08502 in patients with advanced solid tumors [76]. It has been suggested that the compound’s pan-CLK inhibitory activity is substantial for sufficient in cell and in vivo anti-tumor activity [76]. Particularly, it seems to be important that SM08502 inhibits also CLK3, which is often not the case for other CLK inhibitors [76].

**Figure 28:**
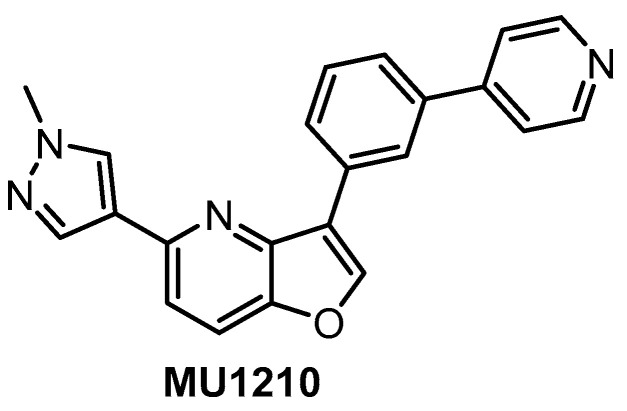
Structure of the compound MU1210.

In vitro profiling of a series of 3,5-disubstituted furo[3,2-*b*]pyridines revealed highly potent and selective CLK1/2/4 inhibitors [47]. The analog most active in the cell, compound MU1210 (Figure 28), was profiled in greater detail using radioenzymatic and NanoBRET assays, in parallel with the negative control MU140 (Table 4).

HIPK2 was revealed as the most significant off-target in vitro (biochemical assay); however, it was not inhibited by MU1210 in the cell (based on the NanoBRET assay). Similarly, the NanoBRET assay demonstrated only very weak activity against DYRKs (typical problematic off-targets of CLK inhibitors) and GSK3α, and overall excellent selectivity profile in the cell. Additional weak off-targets determined in the primary biochemical screen were GSK3α, PIM1, PIM2, and HASPIN exhibiting residual activity of 10%, 16%, 19%, and 26%, respectively, at 1 µM concentration of MU1210. MU1210 exhibited anti-tumor activity in MCF7, MDA MB 231, and MCF10A breast cancer cell lines (with EC_50_ values of 1.1, 1.3, and 1.5 µM, respectively) as well as in HeLa, U2OS, PANC1, HEK293T, AGP01, and MEF cancer cell lines. In addition, MU1210 affected phosphorylation of SR protein in HeLa cells (while the negative control MU140 had no effect) and modulated alternative mRNA splicing of *MDM4* [47,152].

Overall, the compound MU1210 is a potent CLK1/2/4 inhibitor in vitro as well as in the cell that exhibits excellent selectivity profile across the kinome, fulfilling the criteria for a quality chemical probe [154,155,156]. The compound has been recommended by the Structural Genomic Consortium as the state-of-the-art tool compound for CLK1/2/4 [152].

**Figure 29:**
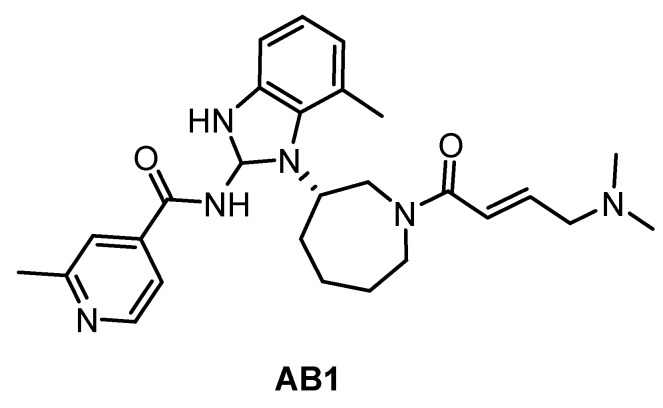
Structure of the compound AB1.

The irreversible inhibitor of *Trypanosoma brucei* CLK1, compound AB1 (Figure 29), was published very recently [89]. The phenotypic screen of the *T. brucei* bloodstream forms with the Novartis kinase-focused inhibitor library (2.3 million compounds) identified compounds with pan-kinetoplastid activity. Further testing against *T. brucei* mutants that overexpress known essential protein kinases revealed CLK1 as the primary target and subsequent SAR studies provided the optimized compound AB1. The irreversible competitive inhibition of CLK1 is provided by the Michael acceptor acryl amide motif, which forms a covalent bond with the C215 residue in the active site cavity. That residue is not present in human CLK1, thereby providing the compound’s selectivity for *Trypanosoma brucei* CLK1 over the human variant. AB1 exhibits also potent inhibitory activity against hERGFR and BTK kinases (IC_50_ values are 2 nM and 2 nM, respectively); additional information on the selectivity has not been published yet [89].

**Figure 30:**
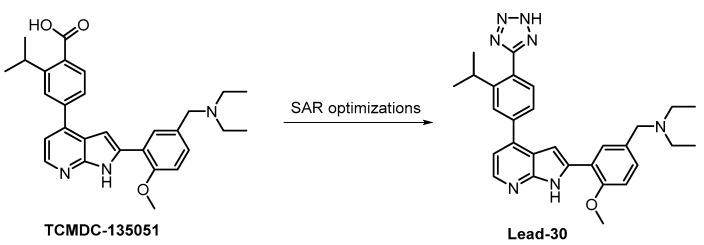
Structures of the compounds TCMDC-135051 and Lead-30.

The compound Lead-30 as well as its precursor, the screening hit TCMDC-135051 (Figure 30), were published very recently [130,131]. Both TCMDC-135051 and Lead-30 inhibit *Pf*CLK3 in *Plasmodium falciparum* (IC_50_ = 40 nM and 19 nM, respectively). Screening of TCMDC-135051 (at 1 µM concentration) against 140 kinases revealed 14 additional kinases showing residual activity below 20%. TCMDC-135051 was found to be also active in vivo—its dose-dependent efficacy was demonstrated in *P. berghei*-infected mice [130]. These results make TCMDC-135051 and Lead-30 good candidates for further development; however, the compounds have not been sufficiently profiled for selectivity to be utilized as quality chemical probes.

**Figure 31:**
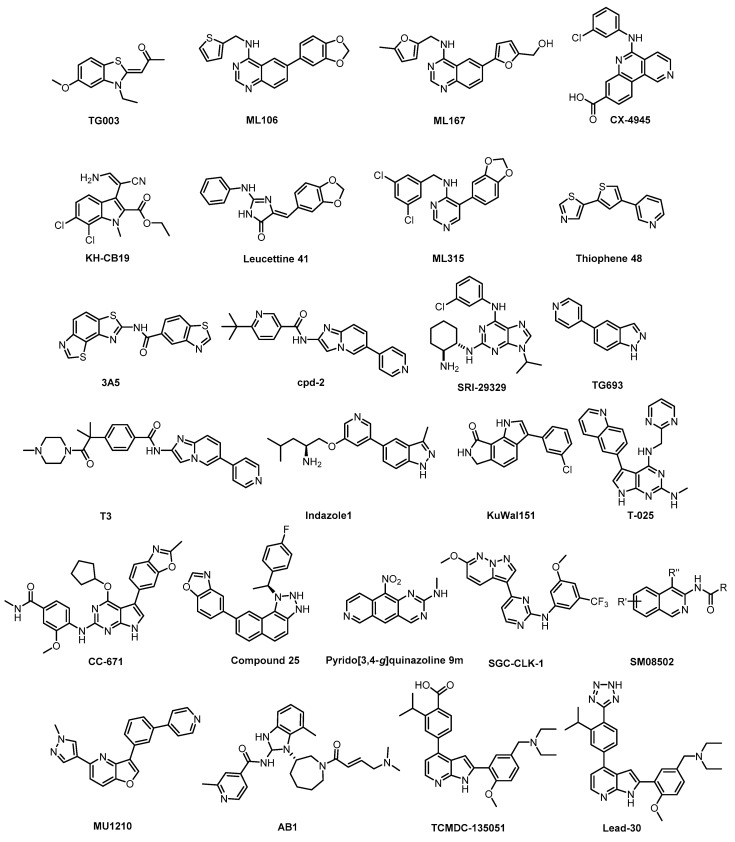
Chemical structures of the reviewed CLK inhibitors.

The chemical structures of the CLK inhibitors reviewed above are collectively represented in Figure 31.

## 7. Conclusions

Over the last two decades, a growing number of studies not only elucidated various aspects of the relatively complex biology of CLKs but also pre-clinically investigated their therapeutic potential. Some of the recently discovered structurally diverse small-molecule CLK inhibitors (summarized in this review) demonstrate sufficient selective activity to fulfill the criteria for quality chemical biology probes. The use of those selective compounds will very likely help further investigate different aspects of the CLK biology, including the role of the individual isoforms and therapeutic potential of their specific inhibition, which may be important in some contexts. The first CLK inhibitor SM08502 that entered clinical trials quite recently (and other compounds that are likely to follow) will demonstrate whether and how inhibition of CLKs can be used for the development of novel medications.

## Figures and Tables

**Table 1:** Human CLKs: amino acid residues count, mass in kDa, and amino acids present in the ATP phosphates’ binding region (the residues specific for individual isoform are in bold) [38].

CLK Form (*Uniprot* Ref.)	Amino Acid Residues	kDa	Amino Acids in the ATP Phosphates’ Binding Region
**CLK1** (P49759)	484	57	167–175 (LGEG**A**FG**K**V)
**CLK2** (P49760)	499	60	169–177 (LGEG**T**FG**R**V)
**CLK3** (P49761)	638	73	310–318 (LGEG**T**FG**K**V)
**CLK4** (Q9HAZ1)	481	57	165–173 (LGEG**A**FG**K**V)

**Table 3:** K_d_ values for kinases significantly inhibited by Leucettine 41 at 10 µM concentration [142].

Kinases	Kd (nM)
CLK4	70.0
DYRK1A	7.8
CLK1	75.0
DYRK2	450.0
HIPK1	320.0
CLK3	1100.0
IRAK1	930.0
HIPK3	230.0
DYRK1B	140.0
CLK2	360.0
TAOK1	1700.0
TYK2	n.t
GSK3A	550.0

**Table 4:** Biochemical and cellular activity of MU1210 and negative control MU140. The cellular K_i_ values were determined by NanoBRET assays in HEK293T cells.

Kinase	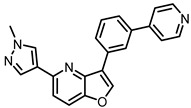 MU1210 Activity [nM]		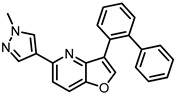 MU140 Activity [nM] (Negative Control)	
	Biochemical IC_50_	Cellular K_i_	Biochemical IC_50_	Cellular K_i_
CLK1	8	84	>3000	>10,000
CLK2	20	91	>10,000	>10,000
CLK3	>3000	-	>3000	-
CLK4	12	23	>10,000	>10,000
DYRK1A	213	6580	>3000	>10,000
DYRK1B	956	>10,000	>3000	>10,000
DYRK2	1309	1700	>10,000	>10,000
HIPK1	187	-	>10,000	-
HIPK2	29	>10,000	>10,000	-
HIPK3	159	-	>3000	-
HIPK4	-	5410	-	-
